# ANGDelMut – a web-based tool for predicting and analyzing functional loss mechanisms of amyotrophic lateral sclerosis-associated angiogenin mutations

**DOI:** 10.12688/f1000research.2-227.v3

**Published:** 2014-02-10

**Authors:** Aditya K Padhi, Suhas V Vasaikar, Bhyravabhotla Jayaram, James Gomes

**Affiliations:** 1 Kusuma School of Biological Sciences, Indian Institute of Technology Delhi, New Delhi, 110016, India; 2 Department of Chemistry, Indian Institute of Technology Delhi, New Delhi, 110016, India; 3 Supercomputing Facility for Bioinformatics and Computational Biology, Indian Institute of Technology Delhi, New Delhi, 110016, India

## Abstract

ANGDelMut is a web-based tool for predicting the functional consequences of missense mutations in the angiogenin (ANG) protein, which is associated with amyotrophic lateral sclerosis (ALS). Missense mutations in ANG result in loss of either ribonucleolytic activity or nuclear translocation activity or both of these functions, and in turn cause ALS. However, no web-based tools are available to predict whether a newly identified ANG mutation will possibly lead to ALS. More importantly, no web-implemented method is currently available to predict the mechanisms of loss-of-function(s) of ANG mutants. In light of this observation, we developed the ANGDelMut web-based tool, which predicts whether an ANG mutation is deleterious or benign. The user selects certain attributes from the input panel, which serves as a query to infer whether a mutant will exhibit loss of ribonucleolytic activity or nuclear translocation activity or whether the overall stability will be affected. The output states whether the mutation is deleterious or benign, and if it is deleterious, gives the possible mechanism(s) of loss-of-function. This web-based tool, freely available at http://bioschool.iitd.ernet.in/DelMut/, is the first of its kind to provide a platform for researchers and clinicians, to infer the functional consequences of ANG mutations and correlate their possible association with ALS ahead of experimental findings.

## Introduction

Amyotrophic lateral sclerosis (ALS) is a rapidly progressive, invariably fatal neurodegenerative disorder that causes the selective destruction of motor neurons, primarily the voluntary muscles. As a result of this degenerative process, most patients usually die from respiratory failure within 3–5 years from the onset of symptoms ^1, 2^. The etiology and mechanisms underlying this debilitating disease are not fully understood, and hence, there is currently no curative or disease-halting therapy for this disorder. Among the genetic factors, about 110 genes have documented association with ALS to date ( http://alsod.iop.kcl.ac.uk and http://bioschool.iitd.ac.in/NeuroDNet/index.php) ^3, 4^. Since its first discovery by Greenway *et al.* ^5^, the *ANG* gene has emerged as one of the most frequently mutated genes found in ALS patients of diverse ethnic groups. A total of 19 missense mutations in ANG have been associated with ALS ^6, 7^. In addition, several rare mutations have also been discovered in the *ANG* gene; however, it is not yet known whether these are instrumental in causing ALS ^7^. The human *ANG* gene encodes a 14.1 kDa long monomeric ANG protein that induces neovascularization, maintains the physiology and health of motor neurons by inducing angiogenesis, stimulates neurite outgrowth and path-finding and protects motor neurons from hypoxia-induced death and hence acts as a neuroprotective factor ^8– 12^. ANG binds to its target cells and undergoes nuclear translocation due to the presence of two functional sites such as the receptor-binding site ( ^60^NKNGNPHREN ^68^) and the nuclear localization signal ( ^29^IMRRRGL ^35^) respectively. Another important function of ANG is the ribonucleolytic activity governed by the catalytic triad residues His13, Lys40 and His114 ^8, 9^. Several reports on laboratory-based functional assay experiments ^9– 12^ and molecular dynamics (MD) simulations ^6, 7, 13^ have shown that loss of either ribonucleolytic activity or nuclear translocation activity or both of these functions due to missense mutations in ANG cause ALS. However, no web-based tool is currently available that can establish the functional consequences of ANG mutations and predict the loss-of-function(s) mechanisms. In light of these observations, we developed and hosted the ANGDelMut web-based tool, available at http://bioschool.iitd.ernet.in/DelMut/, which utilizes a MD simulation-based protocol to capture certain structural and dynamic features of simulated mutant proteins to predict mechanisms of functional loss.

**Figure 1.**
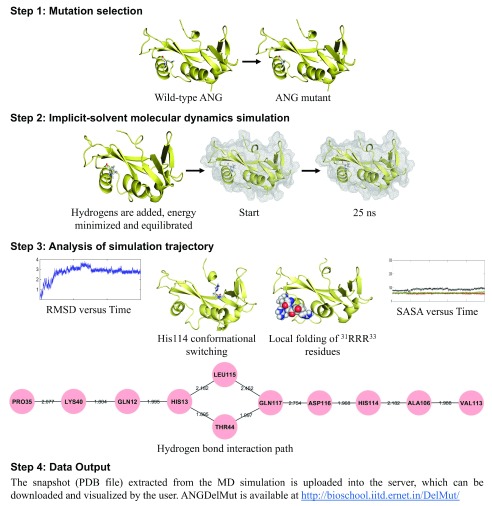
ANGDelMut protocol. Steps involved to predict whether an ANG mutation will be ALS causative or not, with a brief understanding of the mechanism(s) of its loss-of-functions, implemented in the ANGDelMut web-tool. In Step 1, the user submits a missense ANG mutation to the web-tool and the mutated ANG protein is prepared. Next, in Step 2, an implicit-solvent MD simulation for 25 ns is performed after adding hydrogen atoms, and executing energy minimization, equilibration. After completion of the simulation, in Step 3 certain structural and dynamic markers/attributes from the MD simulation trajectory are analyzed, namely, the RMSD versus time, conformational switching of catalytic residue His114, presence of hydrogen bond interaction path from the site of mutation to His114 mediated through Leu115, local folding of nuclear localization signal residues ^31^RRR ^33^ and change in SASA versus time. After analysis in Step 3, the PDB file extracted from the MD simulation and an excel file containing the RMSD data are uploaded into the web-tool.

The method implemented in ANGDelMut has been previously tried and tested, and in addition, the functional loss predictions of mutants have been correlated with known experimental reports ^6, 7, 13^. When a user submits a mutation, the mutant protein is prepared *in silico* by replacing the target residue with the desired amino acid residue. Subsequently, a standard MD simulation-based protocol is followed based on certain optimized parameters, which performs extensive MD simulations for 25 ns. The simulated trajectories are then visualized and analyzed for the presence of certain global attributes ^6, 7, 13^, which in turn indicate whether a mutant will probably exhibit any loss-of-function(s). The output of ANGDelMut suggests the nature of the mutation; it’s probable association with ALS, and further highlights the plausible molecular mechanisms of loss-of-functions to the user ( Figure 1).

## Materials and methods

### Technical details and implementation

The ANGDelMut web-based tool was developed in MySQL and is hosted on an APACHE http server located in the computer service centre of the Indian Institute of Technology, Delhi, India. The following are the minimum hardware and software requirements to run this tool –

- At least 64 MB of available random-access memory (RAM) (256 MB recommended).
- Internet Explorer 7.0 or higher (IE8.0 or higher recommended), Firefox 3.x, Chrome 9, 10.
- JavaScript and cookies enabled, recommend ActiveX enabled for Internet Explorer.
- Flash player installed.

The ANGDelMut algorithm is executed in four steps, namely, mutation selection, implicit-solvent molecular dynamics simulation, analysis of simulation trajectory, and data output ( Figure 1). The source code is freely and permanently available at: 10.5281/zenodo.7478.

***Step 1: Mutation selection.*** In the first step known as “Mutation selection”, the user submits a missense mutation as the input. Once the mutation is submitted, the mutated protein is prepared *in silico* ( Figure 1). The X-ray structure of human angiogenin (PDB code: 1B1I) ^14^ is used as a starting structure to prepare the mutant, from which the crystallographic water and cofactor, citric acid (CIT) is removed while keeping the secondary structure intact.

***Step 2: Implicit-solvent molecular dynamics simulation.*** Before performing MD simulations, the mutated protein is checked for accuracy and missing atoms, if present, are fixed. Following this, hydrogen atoms are added using the Xleap tool of AMBER 11 ^15^ and if required, the system is neutralized with counter ions ( Figure 1). The SANDER module of the AMBER 11 package with the "ff99SB" force field is used for all MD simulations ^16^. A standard protocol for MD simulations is then implemented that consists of an energy minimization (2500 steps of steepest descent followed by 1000 steps of conjugate gradient method), an equilibration phase involving gradual heating from 0 to 300 K in 200 ps followed by a constant temperature equilibration for 1000 ps at 300 K ^13^. Finally, 25 ns production MD simulations are carried out with periodic boundary conditions in the isothermal–isobaric (NPT) ensemble at a temperature of 300 K with Berendsen temperature coupling and a constant pressure of 1 atm with isotropic molecule-based scaling ^17^. The commonly used SHAKE algorithm and particle-mesh Ewald (PME) method are used to constrain bond lengths involving hydrogen atom(s) and in the calculation of long range electrostatic forces, respectively ^18, 19^. An implicit-solvent based MD simulation method which employs the Generalized Born (GB) model to describe the solvation effects implicitly, executes the 25 ns simulation in about 42 hours compared to 625 hours taken by the explicit method. For this, the GB model proposed by Onufriev, Bashford, and Case {generalized Born solvent (igb) = 5)} and the analytical linearized Poisson–Boltzmann (ALPB) approximations are used ^20, 21^. The Born radii were adopted from Bondi with modification (mbondi2). For analysing the simulated trajectories, the trajectory of each system is recorded at every 1 ps. Analysis of simulation results are carried out using PTRAJ module implemented in AMBER 11. All the simulations are performed on a 320 processor SUN Microsystems cluster at the Supercomputing Facility ( http://www.scfbio-iitd.res.in) of the Indian Institute of Technology, Delhi, India. As a quality control measure, each simulation trajectory is visualized using visual molecular dynamics (VMD) ^22^ and monitored by calculating certain parameters, such as, total energy and the root mean square deviation (RMSD) at regular intervals.

***Step 3: Analysis of simulation trajectory.*** Once the simulation is completed, each trajectory is analyzed for the presence of several structural and dynamic attributes. Broadly, these attributes are indicators of overall stability of the mutant, residue-specific conformational changes during the dynamics run, presence of a hydrogen bond interaction path, and changes in solvent-accessible surface area (SASA) values ( Figure 1). The overall stability of the mutant is assessed by calculating the RMSD of the backbone atoms, sampled once every picosecond, using the PTRAJ module of AMBER 11 ^15^ ( Figure 1). Conformational changes of the catalytic triad residues are visualized and monitored using VMD ^22^. After this, the hydrogen bond interaction paths are computed using UCSF CHIMERA ^23^ and visualized using Cytoscape ^24^ to examine the ribonucleolytic activity. The nuclear translocation activity of the submitted ANG mutant is examined by calculating the SASA of nuclear localization signal residues ^31^RRR ^33^ using VolArea ( http://www2.fc.up.pt/PortoBioComp/Software/Volarea/Home.html), a VMD plug-in.

***Step 4: Data output.*** The output panel shows whether the user-submitted ANG mutation exhibited conformational switching of His114 and if it possessed a hydrogen bond interaction path mediated through Leu115, to predict the loss of ribonucleolytic activity. Next, it is determined if the mutated protein exhibited reduction in SASA and local folding of nuclear localization signal residues ^31^RRR ^33^ to predict loss of nuclear translocation activity. Finally, the back bone RMSD data is examined to evaluate its stability ( Figure 1). In addition to all of this information, a Protein Data Bank (PDB) file from the simulation trajectory, a PyMOL .pse session file highlighting the regions of interest and the backbone RMSD data computed from the simulated trajectory in the form of an excel file is made available to the user for downloading, visualization and analyses. Moreover, the functional loss mechanism data interpreted by the host is also suggested and the user is notified via an e-mail, which includes (І) information on time spent by the mutant out of 25 ns in the local conformation(s), (ІІ) threshold values used to predict whether a mutation will exhibit any functional loss, and (ІІІ) an approximate time for the completion of the submitted job.

## Results and discussion

### Features of ANGDelMut

The main interface of ANGDelMut provides the user with a brief description about the tool, how mutations in ANG cause ALS and web links to certain important related articles. Further, the interface has an information panel that gives a brief overview of the methodology employed in ANGDelMut and the various stages involved in query processing and in analysing and obtaining the output data. To submit a mutation, the user selects the source amino acid residue which needs to be mutated with the desired residue. In order to understand the predicted functional loss mechanisms of mutants, the user picks certain attributes from the input panel, such as {Conformational switching of His114, Hydrogen bond interaction path mediated through Leu115 - for loss of ribonucleolytic activity}, {Reduction of SASA, Local folding of nuclear localization signal residues ^31^RRR ^33^ - for loss of nuclear translocation activity} and {RMSD - for stability}, which are analyzed from the simulation trajectory to predict whether a mutation is deleterious or benign. Once all the information is uploaded, the job is manually submitted to the server and the status “Query in queue” is displayed on the output page. In addition, the host is notified by a confirmatory e-mail and with the corresponding Job ID-used to track the job. After checking the accuracy of the input data, the web-tool starts processing the ANG mutant protein and performs the simulations. To enable the user to track the query progress, a progress bar is displayed that shows the completion of each step linked to the user’s Email-ID or Job-ID. Once the job is completed, the results are manually linked to the output page. The user can download the output file, which is a PDB file that contains structural and dynamic attributes. In addition, a PyMOL .pse session file showing the regions of interest and an excel file containing the backbone RMSD data computed from the simulated trajectory are made available to download. Further, the user is notified through an e-mail, once the result is available. The detailed working principle of ANGDelMut, illustrated using two ANG mutations, K17I (found in ALS patients, and known to cause loss of ribonucleolytic activity) ^6, 7, 13^ and L35P (not yet identified in ALS patients but predicted to cause loss of both ribonucleolytic activity and nuclear translocation activity) ^6, 7, 13^ and WT-ANG as control, according to the four steps ( Figure 1) is described below.

### Case study: Prediction of functional loss mechanisms of K17I and L35P-ANG mutants

***K17I and L35P mutant preparation.*** When the user submits an ANG mutation, for example K17I and/or L35P, the PyMOL molecular graphics system ^25^ is used to mutate residue K17 to I17 and L35 to P35 in the crystal structure of ANG (PDB ID: 1B1I without the heteroatoms) and the modified file is then saved in PDB format ( Figure 1). For WT-ANG, only the heteroatoms are removed from the structure and saved in PDB format. Before setting up the simulations, the PDB file of the mutant is inspected to check whether the mutation has affected its secondary structure. We noticed that before simulations, the K17I and L35P mutant had structurally aligned well with the WT-ANG.

**Figure 2.**
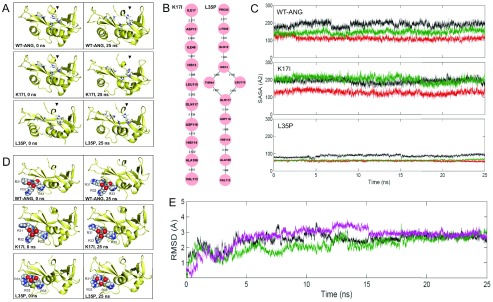
Output results obtained from ANGDelMut for K17I and L35P-ANG mutants. ( **A**) Snapshots of ANG proteins obtained from 25 ns simulation trajectories, showing conformational switching of catalytic residue His114 in K17I- and L35P-ANG mutants compared to WT-ANG, resulting in loss of ribonucleolytic activity. ( **B**) Obtained conserved hydrogen bond interaction paths from the site of mutations Ile17 and Pro35 to catalytic residue His114 from simulations for K17I and L35P mutants, where node represents the amino acid residue and edge represents the bond length between them. These paths mediated through Leu115, which induces in loss of ribonucleolytic activity. ( **C**) Computed SASA values of nuclear localization signal residues ^31^RRR ^33^ from simulations for WT-ANG and K17I and L35P-ANG mutants. SASA values are represented as R31: black, R32: green and R33: red coloured lines. SASA values for WT-ANG and K17I are higher compared to L35P-ANG mutant, suggesting loss of nuclear translocation activity of L35P mutant due to reduction of SASA value. ( **D**) Snapshots of ANG proteins obtained from 25 ns simulation trajectories, showing loose packing of ^31^RRR ^33^ nuclear localization signal residues in WT-ANG and the K17I-ANG mutant suggesting no loss of nuclear translocation activity while in L35P, the ^31^RRR ^33^ residues are in closely packed resulting in reduction in SASA values causing loss of nuclear translocation activity. ( **E**) Plot showing the stability of WT-ANG and K17I-, L35P-ANG mutants during the simulations. The RMSD of the backbone atoms from the equilibrated conformation (0 ns) is presented as a function of time. The RMSD time profiles for WT-ANG, K17I and L35P are shown in black, magenta and green coloured lines respectively.

***Performing implicit-solvent molecular dynamics simulation and analysing the trajectory.*** The K17I, L35P mutants and WT-ANG prepared from the above step were subjected to implicit-solvent MD simulations for 25 ns ( Figure 1). The trajectories of the K17I and L35P mutants and WT-ANG were then visualized using VMD ^22^. First, the ribonucleolytic activity was investigated. We had earlier established that the conformational switching of catalytic residue His114 is responsible for loss of ribonucleolytic activity of certain ANG mutants, and the predictions were correlated with experimental reports ^6, 7, 13^. The whole trajectories of the K17I and L35P mutants, and WT-ANG were scanned and it was observed that the conformation of catalytic residue His114 changed significantly in the K17I and L35P mutants, while the WT-ANG did not show any His114 conformational alterations ( Figure 2A). Further, it was observed that although the site of mutation (Ile17 and Pro35) is distal from the catalytic residue His114, this affected the His114 conformation. In our previous reports, we have determined that in certain mutants, a conserved hydrogen bond interaction path mediated through Leu115 is responsible for conformational switching of His114 ^6, 7, 13^. The hydrogen bond interactions among pairs of amino acid residues were computed from the MD trajectory using UCSF Chimera ^23^ based on a distance cut-off ≤ 3.2 Å and visualized using Cytoscape ^24^. It was found that the K17I mutant possessed a hydrogen bond interaction path Ile17-Asp15-Ile46-His13-Leu115-Gln117-Asp116-His114 while the L35P mutant possessed a Pro35-Lys40-Gln12-His13-(Thr44 and Leu115)-Gln117-Asp116-His114 path, which is responsible for the observed conformational change of His114 ( Figure 2B). The two attributes, such as conformational switching of His114 and presence of a conserved hydrogen bond interaction path mediated through Leu115, derived from the simulation data suggest that the K17I and L35P mutants exhibit loss of ribonucleolytic activity while WT-ANG retained this activity ( Figure 2).

The nuclear translocation activity of K17I and L35P mutants was also studied. Earlier studies conducted by us have established that local folding and close packing of nuclear localization signal residues ^31^RRR ^33^ are responsible for loss of nuclear translocation activity of ANG mutants ^6, 7, 13^. As a result of this local folding, the three successive arginine residues, ^31^RRR ^33^, exhibit a reduction in the SASA value. For the K17I, L35P mutants and WT-ANG, SASA of ^31^RRR ^33^ residues was calculated from their respective MD trajectories. It was observed that L35P had a reduction in SASA compared to WT-ANG and the K17I mutant, as a result of local folding and close packing of nuclear localization signal residues ^31^RRR ^33^ ( Figure 2C and 2D). The two attributes, such as local folding of nuclear localization signal residues ^31^RRR ^33^ and reduction of SASA, derived from the simulation data suggest that the L35P mutant will exhibit loss of nuclear translocation activity while WT-ANG and K17I mutant will retain this activity ( Figure 2).

The overall stability of the K17I and L35P mutants and WT-ANG was studied by calculating the backbone RMSD values from the MD simulations. It was observed that the RMSD value of the K17I and L35P mutant was comparable to WT-ANG, suggesting the mutation from Lys17 to Ile17 and Leu35 to Pro35 did not affected the overall stability of the K17I and L35P mutants during the simulations ( Figure 2E). It is also important to notice that due to such minor alterations in conformations of certain residues, such as the His114 and ^31^RRR ^33^, the overall stability of the mutant remained unaltered.

***Data output.*** The K17I mutant exhibited loss of ribonucleolytic activity due to conformational switching of catalytic residue His114 and presence of a conserved hydrogen bond interaction path mediated through Leu115 while retaining its nuclear translocation activity ( Figure 2). However, the L35P mutant exhibited loss of both ribonucleolytic activity and nuclear translocation activity ( Figure 2). Although the L35P mutant has not yet been identified in ALS patients ^6, 7, 13^, from the simulation results using the ANGDelMut web-tool, it seems that L35P would possibly be a deleterious mutation. The PDB files of the K17I and L35P mutants available for download and visualization show the presence of the above attributes and show the probable mechanisms for the loss-of-functions. The user also gets an excel file containing the backbone RMSD values for K17I and L35P mutants, which can be downloaded and analyzed by the user in order to infer the overall stability of the mutants. Broadly, the users get to know whether the mutation will likely to play a role in ALS pathogenesis. A notification e-mail is also sent to the user informing them of the completion of job and the final output of the query.

Further, it is important to note that missense mutations do not always affect the protein properties (protein structure and conformations); rather these occasionally influence the pre-mRNA splicing process. Therefore, even if MD simulations followed by experiments detect no structural and dynamic changes in protein properties due to mutations, certain nucleotide change may act on other aspects of gene expression such as the mRNA processing.

## Conclusion

ANGDelMut is an easy-to-use web-based tool that incorporates a methodology to predict whether an ANG mutation will be possibly associated with ALS. A set of global attributes are usually investigated from the MD simulations to predict whether a mutation will be deleterious or benign ^6, 7, 13^. Therefore, it is the first tool of its kind to provide the user with details of the probable mechanisms of functional loss of an ANG mutant. The user can get this information relatively quickly ahead of experiments. We hope that the ANGDelMut web-tool, freely available at http://bioschool.iitd.ernet.in/DelMut/, will help clinicians and researchers to understand the pathogenesis and progression of ALS, due to an increase in the number of newly discovered ANG mutations.
