# Supplementary material for: ANGDelMut – a web-based tool for predicting and analyzing functional loss mechanisms of amyotrophic lateral sclerosis-associated angiogenin mutations
Source: F1000Res. 2014 Feb 10;2:227. Originally published 2013 Oct 28. [Version 3] doi: 10.12688/f1000research.2-227.v3 (PMC3901456; doi:10.12688/f1000research.2-227.v3)
Supplement: Peer review history

# Peer review: Referee response for version 3

**DOI:** 10.5256/f1000research.3845.r3592

**Published:** 2014-02-11

**Authors:** Emanuele Buratti

**Affiliations:** 1 Molecular Pathology, International Center for Genetic Engineering and Biotechnology, Trieste, Italy

The authors have introduced all the required changes.

I have read this submission. I believe that I have an appropriate level of expertise to confirm that it is of an acceptable scientific standard.

# Peer review: Referee response for version 2

**DOI:** 10.5256/f1000research.3396.r3017

**Published:** 2014-01-22

**Authors:** Emanuele Buratti

**Affiliations:** 1 Molecular Pathology, International Center for Genetic Engineering and Biotechnology, Trieste, Italy

The ANGDelMut tool is one of the latest additions to the long list of web-based tools that aim to provide researchers with a preliminary possibility to screen for pathogenic mutations in a gene/protein of interest. In this particular case, the authors have focused on mutations occurring in the Angiogenin gene that was previously found to be associated with the development of Amyotrophic Lateral Sclerosis in several patients. Overall, I found the Introduction well written and comprehensively explained, especially after the first round of revision.

As with many tools of its kind, in my opinion two factors are very important to meet user expectations: "friendliness" of the interface and speed of execution.

With regards to the interface I found it rather easy to use. It is possible to set up a Batch job within minutes of having seen it for the first time, and I was notified by e-mail of its acceptance immediately (for some reason I received two identical mails, but this is a minor glitch).

First of all, something that I noticed is that in my first query the answer I received after a few hours was the following "... your submitted mutation is located at the signal peptide region of Angiogenin. In other words, the source residue which has to be mutated, is not present in the mature protein and thus is absent in the crystal structure (3D structure)". I can of course understand the reason for this, but I also wonder why, in the first place, there is the possibility to search for mutations in regions where the authors already know that no prediction will be made. As far as I could see, this has not been mentioned in the program interface, and should be clearly stated (alternatively, I would make it impossible to search in the signal peptide region).

Regarding the speed of execution, after 24 hours of setting up my second query (outside the signal peptide region) the job was 70% to completion according to the status bar (that I found a very good addition to the program). Whilst I found this an acceptable time, as we are talking about MD simulations, I also have to note that in this query I only specified the attribute for loss of ribonucleolytic activity. This probably means that readers should be well advised not to make a "blanket" search for all the possible attributes, unless they are prepared to wait for a very long time. It would be great, although I am not sure if feasible, if users could receive a rough idea of the time they will have to wait to receive an answer, depending on the options chosen.

In conclusion, my experience with the interface was rather positive and I experienced no problems whatsoever with serve connection etc... Therefore, although I also would like to stress that especially in a clinical setting predictions are pretty much useless in the absence of experimental validation, my opinion is that ANGDelMut is a very ambitious, and mostly successful approach to try and bring the world of MD simulations to researchers who do not have advanced training in this highly specialized field. At the very least, ANGDelMut would probably be useful to "prioritize" experimental validations in the presence of several mutations to be tested.

Finally, as a general note, readers should also be made aware that missense mutations at the aminoacid level may not necessarily be acting just on the protein properties but also on the pre-mRNA splicing process (samesense mutations, of course, can do this as well). In this particular case, the ANG gene is composed of only two exons with the entire coding region contained in the second and last exon (that, like first exons, is subject to some rather distinct recognition models with respect to classical internal exons). Therefore, I think it would be well if the authors could also add in the Discussion that even if MD simulations and experimental validations detect no change in protein properties it is always possible that a particular nucleotide change may act on other aspects of gene expression such as mRNA processing.

I have read this submission. I believe that I have an appropriate level of expertise to confirm that it is of an acceptable scientific standard, however I have significant reservations, as outlined above.

# Response

**Published:** 2014-02-07

**Authors:** James Gomes

**Affiliations:** Indian Institute of Technology Delhi, India

We thank the reviewer for appreciating our work and for his positive, constructive comments about the ANGDelMut web-tool.

- As per the reviewer’s suggestion, we have removed the amino acid residues associated with the signal peptide region of Angiogenin from the ANGDelMut interface. Hence, the user can submit mutations present in the mature Angiogenin protein only. 
- We appreciate the viewpoint of the reviewer about notifying the user an approximate time by which the job will be completed. We have prepared a process flow with regards to the time for each step and from now onwards, the user will receive an estimate of the time required for completion in the reply e-mail.
- We agree with the reviewer that experimental validation of the predictions for loss-of-functions is desirable. Although we have not carried out any experiments, our predictions were validated against published results ( Padhi *et al., *2012; Padhi *et al.*, 2013; Padhi *et al., *2013 ). We hope that ANGDelMut will useful to researchers and clinicians working in this field and find wider acceptance.
- We have incorporated a sub-section in the Discussion as suggested by the reviewer.

# Peer review: Referee response for version 2

**DOI:** 10.5256/f1000research.3396.r2890

**Published:** 2014-01-03

**Authors:** Rafael Najmanovich

**Affiliations:** 1 Department of Biochemistry, Université de Sherbrooke, Sherbrooke, Canada

In my opinion the authors addressed only minor points, and still did not address satisfactorily my main two requests. Namely, to clearly highlight:

1. The fact that their proposed mechanism for loss of functionality is not experimentally validated in any way, even if their predictions correlate with experiments in some cases.
2. All steps in their hybrid human/web tool where human intervention are required, be it in data preparations, visualization and analysis.

I have read this submission. I believe that I have an appropriate level of expertise to confirm that it is of an acceptable scientific standard, however I have significant reservations, as outlined above.

# Peer review: Referee response for version 1

**DOI:** 10.5256/f1000research.2772.r2227

**Published:** 2013-11-25

**Authors:** Rafael Najmanovich

**Affiliations:** 1 Department of Biochemistry, Université de Sherbrooke, Sherbrooke, Canada

The authors have created a web-based tool to predict the effect of mutations on the angiogenin protein that utilizes Molecular Dynamics simulations to evaluate if the selected mutation is benign or otherwise deleterious. I have several criticisms regarding this work both in terms of: 1- The implementation of the web-tool described here and more importantly, 2- The methodology behind the web-tool. These are discussed below:

1. Web-tool implementation and function.
  The web-tool seems to only be accessible online intermittently. On several occasions I could not access it. When I could, using Firefox or Safari browsers in Mac OS 10.9, the interface seemed badly designed, not allowing the visual inspection of the selected potential mutations in a 3D model of the protein. The authors could for example, use Jmol for that. 48 hours after submitting a query I am still waiting for any results. The MD protocol implemented by the authors calls for a 25 ns simulation, in our experience we observed that a 25 ns simulation takes approximately 5 days using 48 cores. I would therefore expect the MD simulations performed by the authors to take less than one day. While the language used in the manuscript is not sufficiently precise, the authors mention that *"each trajectory is analyzed for the presence of several structural and dynamic attributes"*. To me this suggests that  there is possibly manual human intervention in the analysis of each query as part of the results and as such, the method would be impractical unless using mechanisms such as Amazon's Mechanical Turk. I cannot comment further on any potential outputs for the submitted queries as I never obtained a result to my first submitted query.
2. The methodology behind the web-tool
  Despite the authors having published 3 papers in peer-reviewed journals using the same methodology as proposed in this web-tool, the reliability of the method in my opinion remains to be proven. I will proceed here to describe the reasons why. The first publication by the authors ( PLoS ONE 7(2): e32479. doi:10.1371/journal.pone.0032479) analyzes 6 mutations (K17I, S28N, P112L, L35P, K60E and V113I). In this work, the authors observe computationally several changes. Notably a conformational change of the catalytic H114 residue side chain and decrease in solvent accessible surface area of the ^31^RRR ^33 ^ signal residues.  Later ( Scientific Reports, January 2013, doi:10.1038/srep01225), the authors considerably expanded their analysis to other mutations and predicted the effect of several other mutations entirely (I46V, K17E, R31K)  or partially (R121H and K54E). This was followed by a third paper ( FEBS Letters, Volume 587, Issue 12, 19 June 2013, Pages 1762–1766 doi: 10.1016/j.febslet.2013.04.022) where some other, rarer mutations are analyzed using the methodology. While the authors present compelling computational results in all these three papers, these results should be viewed as a hypothesis for the mechanism by which such mutations lead to loss of activity. These mechanisms remains to be proved experimentally. It is important to always keep in mind that correlation does not mean causation. As these suggested mechanisms have not been proven to cause the loss of function, their correlation with loss-of-function cannot be used to for ‘predicting and analyzing functional loss mechanisms of deleterious angiogenin mutations casing amyotrophic lateral sclerosis”.

In summary, I have several reservations regarding the usability of the web-tool presented in this manuscript and I object to the use of the mechanisms suggested by the MD simulations, no matter how compelling their likelihood based on computational evidence, as if they were accepted mechanisms without having ever been experimentally validated. The fact that the authors have already published three papers in which the reviewers did not seem to point out this issue, makes this issue all the more important.

I have read this submission. I believe that I have an appropriate level of expertise to state that I do not consider it to be of an acceptable scientific standard, for reasons outlined above.

# Response

**Published:** 2013-11-26

**Authors:** James Gomes

**Affiliations:** Indian Institute of Technology Delhi, India

To the best of our knowledge, the web-tool always opens from different browsers and works fine in different operating systems such as, Windows and Mac. We do not know why the reviewer was unable to access it because during this period our servers were not down. The Jmol JAVA visualizer needs a PDB file for 3D visualization. If a mutation is incorporated, the new coordinates of the C, N, H, O-atoms have to be updated in the PDB file. This is possible only after MD simulations. Therefore, the reviewer’s expectation of a visual inspection of the potential mutations in 3D model in Jmol before MD simulations is unrealistic. As a part of our output, we provide the user with the PDB file for visualization.

The results of the reviewer submitted query were available within 48 hours after the start of the simulation and are still available on the web server (see here for a screenshot) . However, after manual inspection of the output file for accuracy, completeness and interpreting the results (deleterious or benign mutation), the output files were uploaded to the web-server within the next 12-14 hours and an e-mail was sent to the reviewer informing this. The user was notified of the completion of the job by e-mail (a screenshot of the email is available here). A Word file, named “Reply_JobID-D00018” was also sent as part of the email to his e-mail ID on 22nd November 2013 at 4:31 PM, Indian Standard Time, to provide further information on the interpreted results.

We are not sure how the reviewer got the impression that a 25 ns-simulation could be completed in approximately five days using 48 cores. We would expect the reviewer to be aware that simulation times vary depending on the size of the protein and the processors available for computation. With the computational facility available in our institute, a 25 ns time duration implicit-solvent based MD simulation for a mutant Angiogenin protein takes about 42 hours in 16 processors out of the 320 processor SUN Microsystems clusters (please see the text in the manuscript “An implicit-solvent based MD simulation method which employs the Generalized Born (GB) model to describe the solvation effects implicitly, executes the 25 ns simulation in about 42 hours compared to 625 hours taken by the explicit method” and “All the simulations are performed on a 320 processor SUN Microsystems cluster at the Supercomputing Facility ( http://www.scfbio-iitd.res.in) of the Indian Institute of Technology, Delhi, India). Once the simulations are completed, the RMSD and PDB files are uploaded to the web-server.

As we use two different servers (one where the web-server is located and another, the supercomputing facility, where the queries are transferred and simulations are performed), for initial query processing and data completeness, human intervention is a mandatory step. In addition, after the completion of the simulations, we visualize and analyze the requested query to check for accuracy and to avoid data incompleteness. This was stated clearly in our manuscript. We hope this answers the reviewer’s comment on this issue. If we submit the reviewer’s e-mail ID or Job-ID into the output panel of the web-tool, even at this point, the output result is still visible. 

In response to the reviewer’s comment about the methodology behind the web-tool we would like to state the following. We are not sure that the reviewer has read the *Scientific Reports* (doi: 10.1038/srep01225) and *FEBS Letters* (doi: 10.1016/j.febslet.2013.04.022) manuscripts in detail. In the *Scientific* *Reports* paper, the last paragraph of the Introduction clearly states that “The close match between simulation results and reported experimental data establishes the strength of this method and its application as a predictive tool to determine if an ANG mutation is deleterious or benign, and if deleterious, the probable mechanism of its loss-of-functions”, rather than only expanding the number of mutations. Also, we would like to suggest that the reviewer checks Table 1 in this article, where we have shown that our predictions matched with all the reported experimental data.

In the *FEBS Letters* paper, we have clearly stated in the abstract that “We present here a fast molecular dynamics based method for determining the mechanisms of functional loss caused by mutations, and attributes to ascertain whether a mutation causes ALS”, rather than highlighting the rare mutations. We have employed the same method in this web-tool as well to understand the functional loss mechanisms. As our predictions match with the reported experimental data, it provides a step forward to use this methodology to predict and analyze the functional loss mechanisms for other new mutations and to predict whether they are deleterious or benign. Thus, we are well aware of the fact that “correlation does not mean causation” as stated by the reviewer. At present, no experimental technique exists that can be used to validate these mechanisms, such as conformational changes of residues at the molecular level, only a time dependence dynamics method, such as, MD simulation can give insights into atomic level details.

In response to the reviewer’s final comment, we would like to emphasize that all our previously published papers have been extensively peer reviewed by the respective referees, with some of them even undergoing 3 or 4 rounds of revision. We feel that the reviewer’s comments that call into question the scientific and intellectual ability of peers across different parts of the world will only discourage authors to publish in open, peer-reviewed journals like *F1000Research*.

# Response

**Published:** 2013-11-30

**Authors:** Rafael Najmanovich

**Affiliations:** Department of Biochemistry, Université de Sherbrooke, Canada

I thank the authors for their extensive response to my original review. It seems that some of the points I made were not sufficiently clear. But before I continue, I would like to acknowledge that I did indeed receive the results in an email but only after I had submitted the original report. I waited for as long as I thought reasonable before submitting it and therefore to be complete, my statement should have read that I did not receive any results up to the time of writing my report. 

The results are available online at the time of writing this response. With respect to the PDB file provided, it would be useful if the authors would adhere more strictly to PDB naming nomenclature, in particular, the authors use the 3-letter code WAT for water molecules instead of HOH. It would also be useful if the authors would provide the possibility to download a Pymol .pse file of the MD trajectory where the regions of interest used in their manual analysis are differentially displayed and also include the possibility to download the raw MD trajectory. The only results that are provided online are a PDB file and an RMSD file, the latter very poorly documented. Additionally, the conclusions of their manual (Human) analysis of the results are displayed but no data are given online as to the fraction of time that systems spends out of the 25 ns simulation in any of the local conformations that lead to their proposed mechanism of loss of ribonucleolytic or nuclear translocation activity mechanisms. Additionally the authors should state online as part of the results given what threshold values are used to conclude based on the dynamics that any of these proposed mechanisms are not favoured in the mutant under study. It is clear that even in the wild type such conformations will be present at least a fraction of the time. This again alludes to my criticism of the human interventions in concluding that any of these proposed mechanisms are prominent during the simulation.

Now, the first point about a Jmol visualization, perhaps I didn't express myself clearly enough, I didn't mean to suggest that the Jmol visualization would show the resulting mutation, of course that requires some form of minimization at the very least. What I would like to see in Jmol is the position of the selected mutation (with the original amino acid).

Regarding the speed of MD simulations, it is not relevant as this discussion was part of my original report in trying to quantify when I could expect the results to arrive as no calculation progress report is given. For example, an email could be sent for a query still running with a link to a results page where the percentage to completion is displayed while the results are not available.

The author may rest assured that I do indeed fulfill their expectation that I am aware that simulation times vary depending on the size of the protein and processors available. The 5 days for a 25 ns simulation using 48 cores represents my subjective experience. I thank the authors for clarifying that several steps require manual intervention. I still think that manual intervention in the generation of results (be it transferring files across servers, checking the output file for accuracy, completeness and interpretation of results), as noted in the original report, is neither dependable not scalable and therefore is not acceptable. What will happens in five years when the postdoc or student responsible for the manual intervention leave? Or when they go on vacation next time?

Contrary to what the authors suggest, I did indeed carefully read their three previous published manuscripts before writing my report on this manuscript. The authors cite in their current response some of their conclusions in these previous reports. While the language used in the *Scientific Reports* manuscript is correct, stating 'probable mechanism' in the *FEBS Letters* paper it becomes 'determining the mechanism'. In this manuscript it turns to even more emphatic language saying 'elucidating the mechanisms'. I am glad that the authors are aware that correlation does not imply causation. Irrespective of what experimental techniques can or cannot be used to prove the veracity of the proposed mechanisms, until experimental validation confirms the mechanisms, the proposed mechanisms will remain *proposed* mechanisms only. I encourage the authors to be clear about this and I remain convinced that this point should have been more clearly stated by the authors in the other papers. Unfortunately, precisely because the peer reviews of these other papers are not openly accessible, I cannot judge the quality of the peer review. Nowhere did I call into question the 'scientific and intellectual abilities' of previous peer reviewers. I said that the fact that it had been missed makes emphasizing this issue here all the more important.

As much as the problems with manual intervention are in my mind sufficient to object to the current manuscript, I am willing change my classification to 'accept the with reservations' as long as the manuscript is extensively rewritten, including changing the title to remove any suggestions, implicit or otherwise, that the mechanisms used to classify the mutations are in any way proven as well as to clearly state the limitations of the manual intervention steps. Furthermore, the proposed hybrid Human/Web-tool presented should be modified to give users access to a job progression report page that is frequently updated and that can be accessed at any time after submission until the results are available.

# Response

**Published:** 2013-12-23

**Authors:** James Gomes

**Affiliations:** Indian Institute of Technology Delhi, India

- *"I would like to acknowledge that I did indeed receive the results in an email but only after I had submitted the original report. I waited for as long as I thought reasonable before submitting it and therefore to be complete, my statement should have read that I did not receive any results up to the time of writing my report."*
  Our record shows that the e-mail was sent to the reviewer on 22nd November 2013 at 4:31 PM, Indian Standard Time (as shown in the attached screen shots of our earlier response) and the output result files were available in time.
- *"The results are available online at the time of writing this response. With respect to the PDB file provided, it would be useful if the authors would adhere more strictly to PDB naming nomenclature, in particular, the authors use the 3-letter code WAT for water molecules instead of HOH."*
  Our web-tool uses the AMBER software package for MD simulations and thus the PDB file, which was retrieved from the MD trajectory, has the WAT code for water molecules instead of HOH. As this has no affect in the interpretation and visualization of the output PDB file, we did not replace WAT with HOH.
- *"It would also be useful if the authors would provide the possibility to download a Pymol .pse file of the MD trajectory where the regions of interest used in their manual analysis are differentially displayed and also include the possibility to download the raw MD trajectory. The only results that are provided online are a PDB file and an RMSD file, the latter very poorly documented."*
  We agree with the reviewer that uploading a Pymol .pse session file can help the user in directly visualizing the regions of interest. Therefore, we have now accepted the above suggestions of the reviewer. As the raw MD trajectory files are usually very large in size, it is not possible to provide the user an option of downloading these files. For the reviewer’s comment about the RMSD data, we are now providing an excel file of RMSD for downloading.
- *"Additionally, the conclusions of their manual (Human) analysis of the results are displayed but no data are given online as to the fraction of time that systems spends out of the 25 ns simulation in any of the local conformations that lead to their proposed mechanism of loss of ribonucleolytic or nuclear translocation activity mechanisms. Additionally the authors should state online as part of the results given what threshold values are used to conclude based on the dynamics that any of these proposed mechanisms are not favoured in the mutant under study."*
  As suggested by the reviewer, we are now incorporating the data on the time spent by the mutant out of 25 ns in the local conformations in the file sent to the user through e-mail. We are also adding the threshold values used to predict whether a mutation will exhibit any loss-of-functions.
- *"What I would like to see in Jmol is the position of the selected mutation (with the original amino acid)."*
  We are working on the Jmol visualization at present and will be adding this feature as soon as possible.
- ***"**Regarding the speed of MD simulations, it is not relevant as this discussion was part of my original report in trying to quantify when I could expect the results to arrive as no calculation progress report is given. For example, an email could be sent for a query still running with a link to a results page where the percentage to completion is displayed while the results are not available."*
  We have incorporated a progress bar for the query progression. Once the user submits his/her Email-ID or Job-ID into the Output section of the web-tool, the output displays “Query in queue” or a “progress bar” showing the step number which has been completed.
- *"I thank the authors for clarifying that several steps require manual intervention. I still think that manual intervention in the generation of results (be it transferring files across servers, checking the output file for accuracy, completeness and interpretation of results), as noted in the original report, is neither dependable not scalable and therefore is not acceptable."*
  We agree with the reviewer that a completely automated process flow is desirable. However, our institute LAN does not permit us to access the Supercomputing facility ( http://www.scfbio-iitd.res.in) servers without log-in. Therefore, the two steps – uploading the jobs and downloading the results are inevitably manual. Even if the next version of the web-tool is hosted in the Supercomputing facility, we will need to manually download the result, analyse the trajectory and link it to the output page of the web-tool.
- *"The authors cite in their current response some of their conclusions in [their] previous reports. While the language used in the Scientific Reports manuscript is correct, stating 'probable mechanism' in the FEBS Letters paper it becomes 'determining the mechanism'. In this manuscript it turns to even more emphatic language saying 'elucidating the mechanisms'."*
  As per the reviewer’s comments, we have made the necessary changes in this version of our manuscript. We have replaced the word “elucidate” with “predict” and made the required changes as suggested by the reviewer. Regarding validation of the proposed mechanisms, we would again like to emphasize that apart from a time dependence dynamics method, such as MD simulations, no other experimental technique exists at present that can be used to validate these mechanisms. However, if the reviewer reads our previous paper ( Padhi *et al*, 2012), he can note that we have validated the proposed mechanism using proper computational control. We suggest the reviewer reads the “MD Simulations” subsection of the “Results” section - “We also validated the 50 ns period by performing a simulation of……..which was then used for subsequent simulation experiments and analyses”.
- " *As much as the problems with manual intervention are in my mind sufficient to object to the current manuscript, I am willing change my classification to 'accept the with reservations' as long as the manuscript is extensively rewritten, including changing the title to remove any suggestions, implicit or otherwise, that the mechanisms used to classify the mutations are in any way proven as well as to clearly state the limitations of the manual intervention steps. Furthermore, the proposed hybrid Human/Web-tool presented should be modified to give users access to a job progression report page that is frequently updated and that can be accessed at any time after submission until the results are available.*"
  We have addressed the reviewer’s major concerns and modified the title of our manuscript. After reading, we have modified and added text in several sections of the manuscript. We have also indicated that we require manual intervention in providing the output to the user. We hope the reviewer finds this revised version of our manuscript suitable for changing the current status to “Approval”.

# Peer review: Referee response for version 1

**DOI:** 10.5256/f1000research.2772.r2228

**Published:** 2013-11-07

**Authors:** Ammar Al-Chalabi

**Affiliations:** 1 King's College London, London, UK

In this interesting paper, Aditya Padhi and colleagues describe a mechanism for predicting the effect of mutations in the ANG gene on the risk of ALS. The paper is well written, the title is appropriate, the abstract provides a good summary and there is an excellent explanation of the study design, methods and analysis.

I have only two minor recommendations:

First, the authors state that there is no successful therapy for ALS. This completely depends on what is meant by successful; there are many therapies and it is the only neurodegenerative disease for which a disease-modifying therapy exists - riluzole. I agree there are no curative or disease-halting therapies. Perhaps this sentence could be rephrased.

Secondly, the authors state that functional assays have been used to predict whether certain mutations cause ALS. I would argue that this is not possible. They can only show a laboratory-based mechanism that is consistent with ALS causation. 

It would be helpful if the authors could provide the URL for the ALSoD database at first citation. ( http://alsod.iop.kcl.ac.uk).

I have read this submission. I believe that I have an appropriate level of expertise to confirm that it is of an acceptable scientific standard.
